# COVID-19 Symptom-Related Google Searches and Local COVID-19 Incidence in Spain: Correlational Study

**DOI:** 10.2196/23518

**Published:** 2020-12-18

**Authors:** Alberto Jimenez Jimenez, Rosa M Estevez-Reboredo, Miguel A Santed, Victoria Ramos

**Affiliations:** 1 Instituto de Salud Carlos III Information and Communication Technologies Unit Madrid Spain; 2 Instituto de Salud Carlos III National Epidemiology Centre Madrid Spain; 3 Faculty of Psychology Universidad Nacional de Educación a Distancia Madrid Spain; 4 Instituto de Salud Carlos III Telemedicine and Digital Health Research Unit Madrid Spain

**Keywords:** behavioral epidemiology, big data, smart data, tracking, nowcasting, forecast, predict, infosurveillance, infodemiology, COVID-19

## Abstract

**Background:**

COVID-19 is one of the biggest pandemics in human history, along with other disease pandemics, such as the H1N1 influenza A, bubonic plague, and smallpox pandemics. This study is a small contribution that tries to find contrasted formulas to alleviate global suffering and guarantee a more manageable future.

**Objective:**

In this study, a statistical approach was proposed to study the correlation between the incidence of COVID-19 in Spain and search data provided by Google Trends.

**Methods:**

We assessed the linear correlation between Google Trends search data and the data provided by the National Center of Epidemiology in Spain—which is dependent on the Instituto de Salud Carlos III—regarding the number of COVID-19 cases reported with a certain time lag. These data enabled the identification of anticipatory patterns.

**Results:**

In response to the ongoing outbreak, our results demonstrate that by using our correlation test, the evolution of the COVID-19 pandemic can be predicted in Spain up to 11 days in advance.

**Conclusions:**

During the epidemic, Google Trends offers the possibility to preempt health care decisions in real time by tracking people's concerns through their search patterns. This can be of great help given the critical, if not dramatic need for complementary monitoring approaches that work on a population level and inform public health decisions in real time. This study of Google search patterns, which was motivated by the fears of individuals in the face of a pandemic, can be useful in anticipating the development of the pandemic.

## Introduction

### Background

During the 2020 Chinese Lunar New Year, massive measures for reducing the spread of the new COVID-19 disease were first enacted by authorities in China [1]. The first reported case of a SARS-CoV-2 infection appeared in late 2019 [2]. Subsequently, the World Health Organization declared the COVID-19 outbreak as a pandemic on March 11, 2020 [3]. The epidemiological characteristics of COVID-19, such as its high transmissibility capacity, virulence, and presence in asymptomatic carriers or those showing only mild symptoms, have yet to be fully understood. At 7 months after the epidemic outbreak in March 2020, we are close to reaching 1 million deaths worldwide [4].

Spain has the fifth highest number of detected COVID-19 cases in the world, behind the United States, Brazil, Russia, and the United Kingdom [4]. As a consequence, developing a forecasting tool to predict the spread of the epidemic has become critical. This information can help us understand the evolution of COVID-19 and how it affects our health. Furthermore, such information can even be useful in preparing for possible future COVID-19 waves and other pandemics.

### Outbreak Detection 

In a study by Chu and Qureshi [5], they state the following:

Predicting the potential spread of a pandemic like COVID-19 is difficult because we do not have many epidemiological data, such as the transmission mechanism, the contagiousness of the virus, or its mutation patterns, as well as other complex human factors, such as the level of compliance with social distancing measures. Many models recently developed by infectious disease scientists [e.g., the Imperial College model [6]…and The Reich Lab [7]…] can produce vastly different predictions as they are constructed based on various assumptions that may not be close to reality (such as the actual level of compliance with social distancing may be much higher than what is assumed in the model, or the infection rates can vary across different regions and groups of people, which cannot be easily captured by any model).

Google Trends offers a new approach for potentially predicting changes in the pandemic by tracking individuals' concerns through their searches. Gunther Eysenbach [8] is a pioneer in terms of conducting studies on the use of Google Trends in health approaches. Furthermore, Ginsberg et al [9] found a high correlation between the pattern of web search queries and the percentage of patients with influenza-like symptoms, thereby confirming that at specific points in time, Google Trends can detect influenza expansion 1 or 2 weeks earlier than the Centers for Disease Control and Prevention.

Within the discipline of behavioral epidemiology, there are articles that study fear in the development of epidemics (eg, Epstein et al study [10]). In our study, behavioral factors were summarized through Google searches and then used as a correlation variable to identify patterns in the evolution of epidemics.

In recent years, the number of different search engines that deal with infodemiology—“which studies the determinants and distribution of health information for public health purposes” [8]—are increasing, and Google Trends is being tested as a useful tool for tracking social trends [11]. During the COVID-19 pandemic, we found 7 articles published from up to May 2020 that raised the possibility of predicting the development of the disease (Table 1).

**Table 1:** Articles that raised the possibility of predicting the development of COVID-19.

Study (Author)	Search engine	Territory	Terms of interest	Time lag
Li et al [12]	Google Trends, Baidu Index, and Sina Weibo Index	China	“Coronavirus” and “Pneumonia”	6-8 days
Husnayain et al [13]	Google Trends	Taiwan	“Handwashing” and “Mask Related Information”	1-3 days
Effenberger et al [14]	Google Trends	China, Republic of Korea, Japan, Iran, Italy, Austria, Germany, United Kingdom, United States, Egypt, Australia, and Brazil	“Coronavirus (Virus)”	11.5 days
Pekoz et al [15]	Google Trends	United States	“Sore Throat,” “Fever,” and Cough	1-2 weeks
Yuan et al [16]	Google Trends	United States	“COVID Pneumonia” and “COVID Heart”	Approximately 12 days
Higgins et al [17]	Google Trends and Baidu Index	China, worldwide data, Italy and Spain, and the US states of New York and Washington	“Shortness of Breath,” “Anosmia,” “Dysgeusia,” “Ageusia,” “Headache,” “Chest Pain,” “Sneezing,” “Diarrhea,” “Fever,” “Cough,” “Nasal Obstruction,” and “Rhinorrhea”	12 days
Lu and Reis [18]	Google Trends	32 countries	“Coronavirus Symptoms,” “Coronavirus Test,” “Fever,” “Cough,” “Coronavirus,” “Runny Nose,” “Dry Cough,” “Sore Throat,” “Chills,” and “Shortness of Breath”	18-22 days

### Objectives

In this study, a statistical approach was proposed in order to assess the correlation between the incidence of COVID-19 in Spain and search data provided by Google Trends. We aimed to determine whether Google Trends data that are collected for searches using many different keywords that the public has entered into Google's internet search engine during the COVID-19 outbreak period can predict the number of cases reported by the National Center of Epidemiology in Spain (Centro Nacional de Epidemiología [CNE]).

## Methods

### Study Design

Our null hypothesis, H0, was as follows: there is no statistically significant relationship between the variables. The proposed alternative hypothesis, H1, was as follows: the obtained correlation coefficient comes from a population whose correlation coefficient is significant.

To achieve the aforementioned objective, we analyzed search data obtained from Google Trends and the official data for the number of daily cases registered by the CNE during the period between February 20, 2020 and May 20, 2020. The rationale behind choosing this time frame for data analysis was that the CNE kept track of the daily cases using a different method until May 20, 2020. The current counting system was implemented on May 11, 2020. The CNE is the government body responsible for collecting and standardizing the data of the 17 autonomous communities that make up the Spanish state. The further analysis of this second dataset has been planned to extend this study.

### Variables for Google Trends Search Terms

We were unable to obtain data for sex and gender from the 2 groups of variables used in this study. These 2 datasets were assessed for any correlations. Our methodology did not include data from explicit participants. Although this may have limited our study results, it allowed for the timely interpretation of data.

Google Trends searches were carried out by looking up terms related to COVID-19 symptoms and synonyms for the term “COVID-19.” Google Trends provides an index of time series data for the volume of queries users have entered into Google within a given geographic area. Google calculates the number of searches for a given term as a proportion of the total number of searches in each location at any given time. These calculations are normalized with a Google Trends relative search volume (RSV) index, which ranges from 0 to 100. An RSV of 100 designates the date with the highest amount of search activity for a given term [19]. In our previous study [20], we established the mathematical formulation of how Google Trends calculates its monthly RSV index for a particular search term.

The following terms were searched on Google Trends: “cansancio,” which translates as “fatigue”; “coronavirus,” “COVID 19,” “covid 19,” and “COVID19”; “diarrea,” which translates as “diarrhea”; “dolor de garganta,” which translates as “sore throat”; “fiebre,” which translates as “fever”; “neumonia,” which translates as “pneumonia” and was searched without an accent due to being more relevant; “perdida de olfato,” which translates as “lost sense of smell” and was also searched without an accent; and “tos,” which translates as “cough.”

Possible spurious terms that Google Trends pointed out in its related queries were eliminated in the search strings by putting the negation operator “-” before the spurious term. The full terms searched, along with links to the original Google Trends search pages and negative queries, were as follows: “cansancio -sociedad” [21]; “coronavirus,” “COVID 19,” “covid 19,” and “covid19” [22]; “diarrea” [23]; “dolor de garganta” [24]; “fiebre” [25]; “neumonia” [26]; “perdita olfato” [27]; and “tos -opensigma -rap” [28]. The full original Google Trends search results for terms related to symptoms of COVID-19 were specific to Spain and the period from February 20 to May 20, 2020.

### Information Provided by the CNE

The CNE is the official Spanish center that collects and centralizes all the epidemiological information in the country. The accuracy of these data mainly depends on the agencies that supply it. In this study, the data mainly come from the autonomous communities that occupy the second administrative level within the Spanish public administration system.

The CNE itself has given warnings about a certain lack of homogenization in the data coming from the sources of origin (ie, the autonomous communities) during the initial period of the pandemic. This has given rise to certain inconsistencies in the data received, which is a problem that we were able to verify ourselves when transforming the aggregate data from the CNE into daily values. Although we obtained 1 negative value, we did not normalize these data, since we believed that they are indeed representative of the state of emergency we have experienced. In addition, even with this evidently erroneous data, the correlations obtained were very good.

The original initial data for the COVID-19 outbreak in Spain were found on the CNE webpage on May 24, 2020, under the tab “Documentación y Datos” [29]. The official COVID-19 data that the CNE offered at that time were aggregate numbers for the following: polymerase chain reaction (PCR)-positive results, the number of people hospitalized, the number of admissions into intensive care units, and the number of deceased persons. This dataset is no longer available, and the dataset in its place presents the results of the second data collection method, which was implemented on May 11, 2020. Our intention is to do research on this dataset in a future study that uses the same methodology as this study.

In the first part of our analysis, we used PCR-confirmed COVID-19 case data, and for the second part of our analysis, we used 4 separate datasets in order to create daily delay graphs for each symptom or searched term.

### Bias, Study Size, and Participants

A potential source of bias may be the choice of Google Trends search terms, as terms for COVID-19 symptoms differ slightly in the Spanish vocabulary. The study sample is representative of the population of Spain, as we restricted the Google Trends data to searches conducted in Spain and the CNE monitors all COVID-19 cases that occur in Spain.

### Statistical Analysis

The Pearson correlation coefficient was used to study the linear relationship between 2 continuous variables (ie, each of the symptoms searched in Google Trends vs the number of daily PCR-positive cases). This is a parametric test that infers its results to be representative of the real population, which makes it necessary for the distribution of the sample to resemble that of the real world to ensure the normality of the data. Therefore, the data, which was drawn randomly from a population whose correlated variables are normally distributed, must be validated. Given that the sample size was <50, the appropriate test for contrasting the goodness of fit to a normal distribution is the Shapiro-Wilk test, in which the null hypothesis implies that the data are normally distributed.

The Pearson correlation coefficient enabled us to understand the intensity and direction of the relationship between variables. It is a symmetric measure; the correlation between x_i_ and y_i_ is the same as the correlation between y_i_ and x_i_.

Time lag correlations were measured to assess whether increases in Google Trends data had a correlation with the evolution of the pandemic. A threshold of *P*<.05 was used to determine statistical significance.

## Results

### Pearson Correlation Analysis and Shapiro-Wilk Test

Tables 2 and 3 present the Pearson correlation coefficients for each of the symptoms categorized in the searches, from the day of the initial search until 21 days later. For each symptom, the day with the highest correlation was noted.

Table 4 shows whether the variables followed a normal distribution.

**Table 2:** Pearson correlation coefficients and *P* values for each of the symptom-related search terms, excluding fatigue. Time lags with respect to COVID-19 incidence data are also presented^a^.

Lag, days^b^	Sore throat		Coronavirus^c^		Fever		Cough		Diarrhea		Pneumonia		Lost sense of smell	
	*r*	*P* value	*r*	*P* value	*r*	*P* value	*r*	*P* value	*r*	*P* value	*r*	*P* value	*r*	*P* value
1	0.4848	<.001	0.5090	<.001	0.4009	<.001	0.4149	<.001	0.5870	<.001	0.6551	<.001	0.6770	<.001
2	0.5549	<.001	0.5730	<.001	0.4770	<.001	0.4964	<.001	0.6446	<.001	0.7163	<.001	0.7151	<.001
3	0.6121	<.001	0.6273	<.001	0.5437	<.001	0.5741	<.001	0.6815	<.001	0.7654	<.001	0.7489	<.001
4	0.6708	<.001	0.6273	<.001	0.5437	<.001	0.5741	<.001	0.6815	<.001	0.7654	<.001	0.7489	<.001
5	0.7369	<.001	0.7256	<.001	0.6771	<.001	0.6991	<.001	0.7743	<.001	0.8544	<.001	0.7847^d^	<.001
6	0.7679	<.001	0.7521	<.001	0.7238	<.001	0.7439	<.001	0.7782	<.001	0.8639	<.001	0.7806	<.001
7	0.8055	<.001	0.7740	<.001	0.7656	<.001	0.7794	<.001	0.7780	<.001	0.8756^d^	<.001	0.7726	<.001
8	0.8358	<.001	0.8118	<.001	0.8100	<.001	0.8201	<.001	0.7922	<.001	0.8593	<.001	0.7319	<.001
9	0.8608	<.001	0.8507	<.001	0.8434	<.001	0.8584	<.001	0.7907	<.001	0.8604	<.001	0.6814	<.001
10	0.8743	<.001	0.8766	<.001	0.8751	<.001	0.8822	<.001	0.8031	<.001	0.8501	<.001	0.6356	<.001
11	0.8799	<.001	0.8999^d^	<.001	0.9086^d^	<.001	0.9015^d^	<.001	0.8117^d^	<.001	0.8585	<.001	0.6111	<.001
12	0.8924^d^	<.001	0.8944	<.001	0.9039	<.001	0.8965	<.001	0.7858	<.001	0.8484	<.001	0.5592	<.001
13	0.8672	<.001	0.8468	<.001	0.8788	<.001	0.8681	<.001	0.7001	<.001	0.8127	<.001	0.4968	<.001
14	0.8279	<.001	0.8065	<.001	0.8319	<.001	0.8296	<.001	0.6326	<.001	0.7668	<.001	0.4419	<.001
15	0.7664	<.001	0.7443	<.001	0.7743	<.001	0.7839	<.001	0.5913	<.001	0.7099	<.001	0.3803	<.001
16	0.7214	<.001	0.6811	<.001	0.7234	<.001	0.7448	<.001	0.5192	<.001	0.6415	<.001	0.3259	.004
17	0.6720	<.001	0.6214	<.001	0.6827	<.001	0.7030	<.001	0.4733	<.001	0.5844	<.001	0.2524	.03
18	0.6093	<.001	0.5517	<.001	0.6330	<.001	0.6654	<.001	0.4271	<.001	0.5467	<.001	0.2053	.08
19	0.5788	<.001	0.4838	<.001	0.5810	<.001	0.6161	<.001	0.3607	<.001	0.5142	<.001	0.1690	.16
20	0.5192	<.001	0.4083	<.001	0.5164	<.001	0.5598	<.001	0.2845	.008	0.4694	<.001	0.1140	.34
21	0.4314	<.001	0.3208	.003	0.4349	<.001	0.4921	<.001	0.1975	.051	0.3812	<.001	0.0630	.60

^a^Pearson correlation coefficients and *P* values for each of the symptoms are based on the comparison between Google Trends searches for the term and daily polymerase chain reaction-positive cases.

^b^This column refers to the days of lag between the 2 variables being compared.

^c^“Coronavirus” refers to searches for the following terms: “coronavirus,” “COVID 19,” “covid 19,” and “COVID19”.

^d^These are the highest correlations for each symptom.

**Table 3:** Pearson correlation coefficients and *P* values for the search term “fatigue.” Time lags with respect to COVID-19 incidence data are also presented^a^.

Lag, days^b^	Fatigue^c^	
	*r*	*P* value
22	0.3926	<.001
23	0.3782	<.001
24	0.3632	.001
25	0.4947	<.001
26	0.5296	<.001
27	0.5171	<.001
28	0.5480	<.001
29	0.5253	<.001
30	0.4720	<.001
31	0.5342	<.001
32	0.5016	<.001
33	0.5427	<.001
34	0.5521	<.001
35	0.5664	<.001
36	0.6350^d^	<.001
37	0.4981	<.001
38	0.4711	<.001
39	0.4388	<.001
40	0.4631	<.001
41	0.4915	<.001
42	0.5325	<.001

^a^Pearson correlation coefficients and *P* values for fatigue are based on the comparison between Google Trends searches for the term and daily polymerase chain reaction-positive cases.

^b^This column refers to the days of lag between the two variables being compared.

^c^The searches for “fatigue” correlated less strongly than the searches for the symptoms in Table 1, but they do show stronger correlations after 36 days. Therefore, we presented these results in a separate table with a different scale of days.

^d^This is the highest correlation with regard to searches for “fatigue.”

**Table 4:** Shapiro-Wilk test for normality. The critical region (.908) for a sample size of 21 and significance level of α=.05 was obtained from the critical values of W_n,a_ for the Shapiro-Wilk test.

Variable	Statistic	*P* value
Coronavirus	.945	.28
Pneumonia	.861	.007
Fever	.946	.29
Cough	.944	.26
Lost sense of smell	.885	.02
Sore throat	.929	.13
Diarrhea	.877	.01
Fatigue	.952	.36

**Figure 1:**
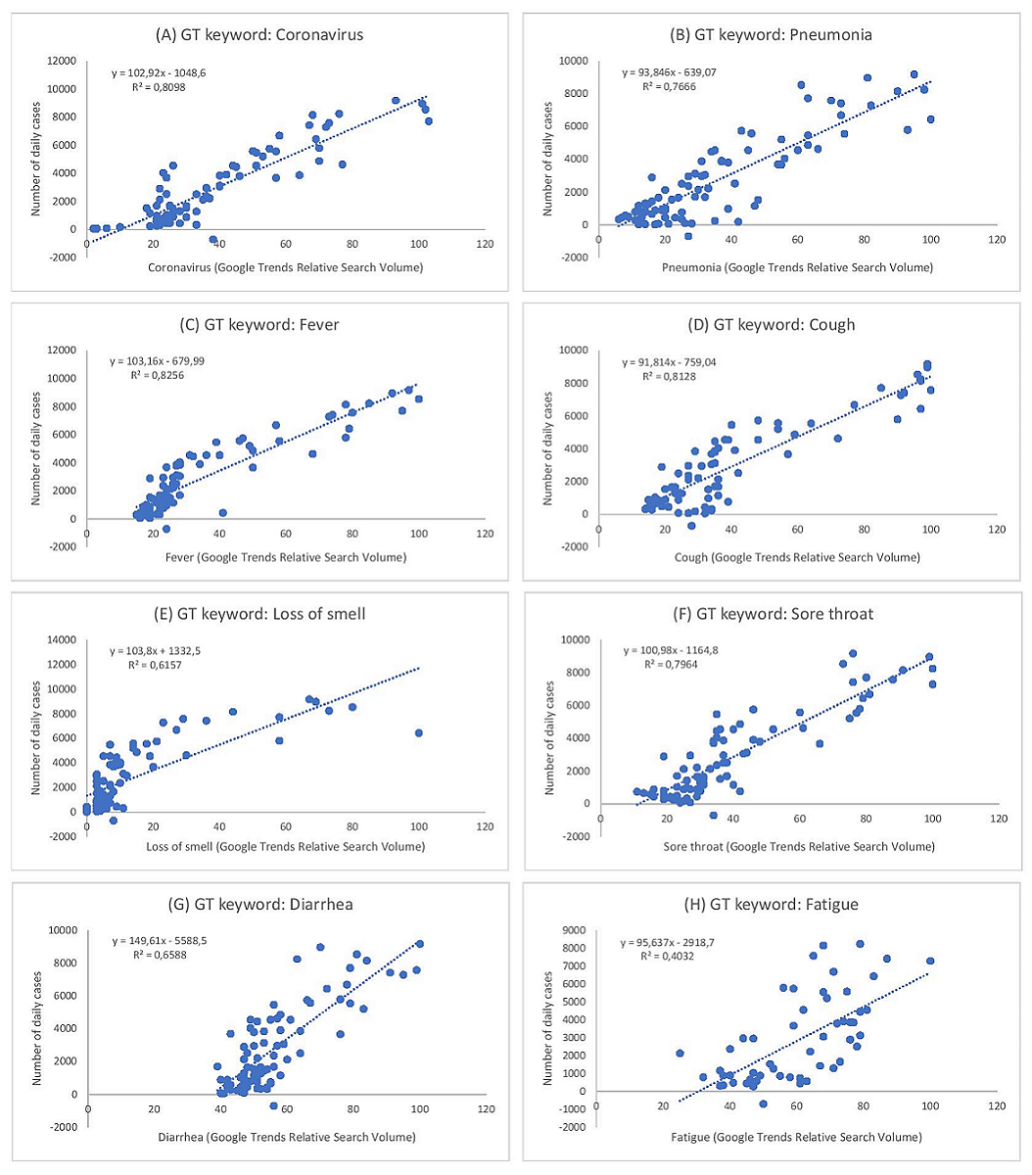
Scatter graphs depicting the linear trend in the relationships between daily PCR-positive confirmed COVID-19 cases and GT searches for COVID-19–related keywords in Spain from February to May, 2020. (A) Scatter graph between daily PCR-positive confirmed COVID-19 cases and GT search term Coronavirus. (B) Scatter graph between daily PCR-positive confirmed COVID-19 cases and GT search term Pneumonia. (C) Scatter graph between daily PCR-positive confirmed COVID-19 cases and GT search term Fever. (D) Scatter graph between daily PCR-positive confirmed COVID-19 cases and GT search term Cough. (E) Scatter graph between daily PCR-positive confirmed COVID-19 cases and GT search term Loss of smell. (F) Scatter graph between daily PCR-positive confirmed COVID-19 cases and GT search term Sore throat. (G) Scatter graph between daily PCR-positive confirmed COVID-19 cases and GT search term Diarrhea. (H) Scatter graph between daily PCR-positive confirmed COVID-19 cases and GT search term Fatigue. GT: Google Trends; PCR: polymerase chain reaction.

**Figure 2:**
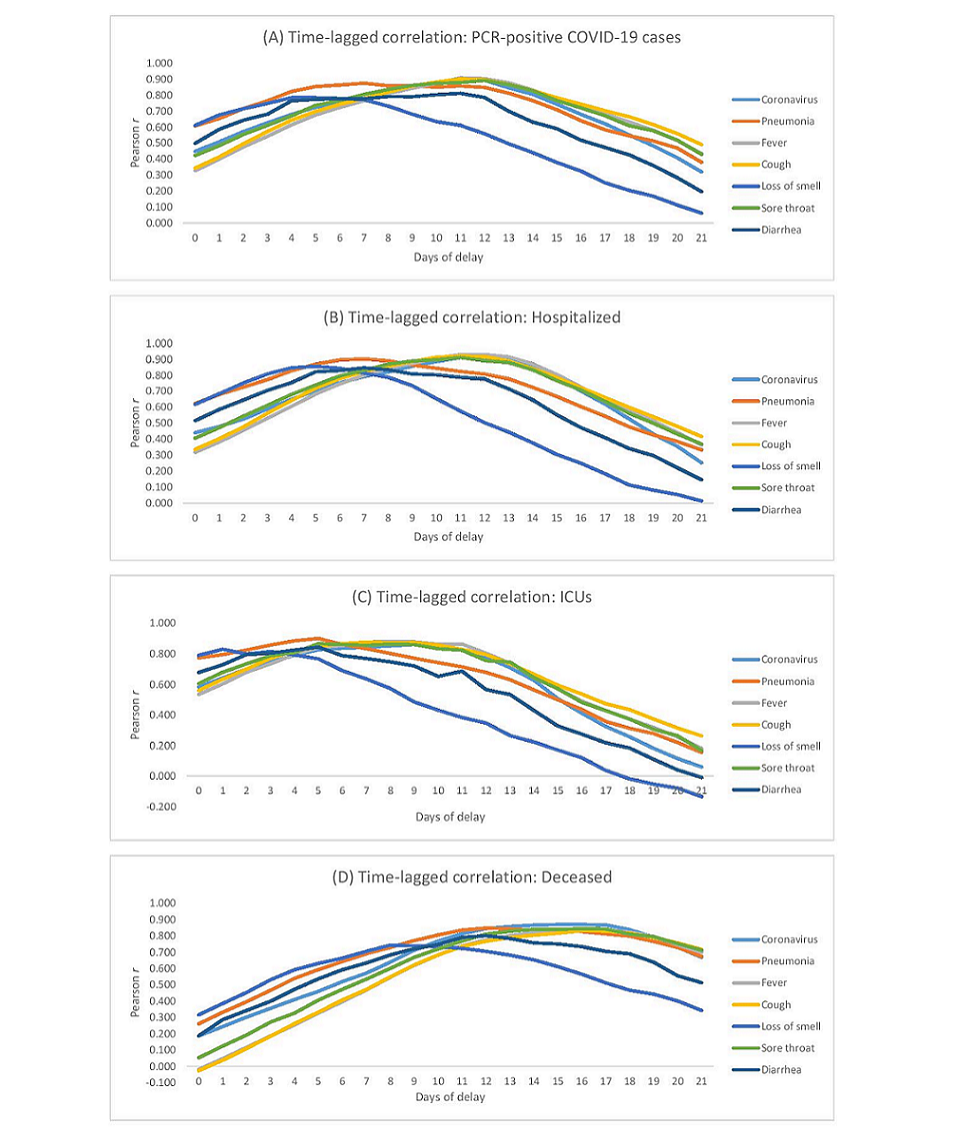
Graphs that show the day with the best correlation between daily PCR-positive COVID-19 cases and GT searches for COVID-19–related keywords. (A) Graph for the comparison between GT search terms and the number of PCR-positive COVID-19 cases, with different day ranges for delay. (B) Graph for the comparison between GT search terms and the number of people hospitalized due to COVID-19, with different day ranges for delay. (C) Graph for the comparison between GT search terms and the number of admissions to the ICU due to COVID-19, with different day ranges for delay. (D) Graph for the comparison between GT search terms and the number of people deceased due to COVID-19, with different day ranges for delay. GT: Google Trends; ICU: intensive care unit; PCR: polymerase chain reaction.

According to Table 4, the variables follow a normal distribution. Based on our results regarding searches for the terms “pneumonia”, “loss of smell,” and “diarrhea,” which have a value lower than the critical region (.908) for the 95% confidence level and a *P* value of <.05, the null hypothesis of normality could be rejected. However, these symptoms do follow a linear trend, as can be in Figure 1. Since these values are close to the critical region, they could also be considered to follow a normal distribution, as seen in Figure 2.

### Outbreak Control Measures

Since the first day of the study period, the number of daily cases began to correlate with searches for all evaluated terms. Since the *P* values ​​for the correlations between daily cases and symptom searches were below the .05 level of significance, it can be stated that the correlation coefficients are significant, which justifies rejecting the null hypothesis. For the majority of the terms that were searched (ie, “coronavirus,” “COVID 19,” “covid 19,” “COVID19”, “fever,” “cough,” and “diarrhea”), day 11 had the highest correlation with the number of new cases.

With regard to searches for “fatigue,” the data began to correlate starting on the third week (ie, day 22). The measures from the previous weeks were eliminated based on the Chauvenet criterion, which states that coefficients outside the confidence interval of 0.3565 and 1.000 can be discarded.

Table 5 shows the coefficients of determination between the number of new cases per day and the rest of the search variables in Google Trends. These coefficients had a critical level of significance (*P*<.001) lower than the established level of significance, which is generally *P*<.05.

**Table 5:** Coefficients of determination between the number of new polymerase chain reaction-confirmed COVID-19 cases per day and the search variables in Google Trends with a critical level of significance (*P*<.001).

Variable	R^2^	*P* value
Coronavirus	0.8098	<.001
Pneumonia	0.7666	<.001
Fever	0.8256	<.001
Cough	0.8128	<.001
Loss of smell	0.6157	<.001
Sore throat	0.7964	<.001
Diarrhea	0.6588	<.001
Fatigue	0.4032	<.001

Table 5 shows that the coefficients of determination were significantly different from 0. Therefore, in this case the null hypothesis is false, as per the Popper methodology [30], and we can affirm that there was a significant positive linear relationship between daily COVID-19 cases and the searches for terms related to COVID-19 and its symptoms on Google Trends. This suggests that the incidence of COVID-19 could be predicted 11 days in advance.

We used graphic procedures to verify the linearity of our results. The graphical representation of the relationship between each term searched and the number of daily cases (Figure 1) shows that there is a linear trend for each relationship. By using the regression lines that were generated, follow-up data can be calculated, including the number of positive cases, thus verifying the correlation between Google Trends searches and the incidence of COVID-19 in Spain.

The graphs in Figure 2 represent the day with the best correlation between both variables (ie, symptom keywords and COVID-19–positive cases). They show the positive relationship between daily cases and the Google Trends searches for terms related to COVID-19 and its symptoms.

### Discussion 

#### Study Implications

Much like the influenza virus, SARS-CoV-2 causes ailments with certain flu-like symptoms, such as cough, fever, and fatigue, and in some cases, these symptoms can complicate differential diagnoses. Examining research that uses nontraditional data sources has several implications. We examined the use of search engines for mitigating the impact of the COVID-19 pandemic. Our results demonstrated that Google can potentially be used as a complementary tool to aid in understanding online search behavior, which could help mitigate the adverse effects of the pandemic and expedite the recovery process.

We found that internet search patterns reveal a robust temporal pattern of disease progression for COVID-19. This study shows that internet search patterns can be used to reveal the detailed clinical course of a disease. These data can be used to track and predict the local spread of COVID-19 before widespread laboratory testing becomes available, and help guide current public health responses.

While laboratory testing serves as an important gauge of epidemic spread, it suffers from a number of important limitations. Alternative surveillance approaches are needed to overcome these limitations and serve as a complement to laboratory testing, especially during the critical early stages of a pandemic. Aggregated deidentified internet search patterns have been used to track a wide range of health phenomena and are a potential alternative source of information for surveilling pandemic spread.

When harnessed appropriately, internet search patterns possess a number of powerful advantages over laboratory testing, such as the following: (1) surveillance data are available immediately when a new pandemic emerges, (2) data are available at a population scale in countries with sufficient internet access, (3) delays are minimal, as search data are available the same day, (4) there is no need for individuals to travel to a testing location; people can stay at home, thereby avoiding increased exposure to other people and health care workers, (5) no physical intervention is required, and (6) the data are available for free, independent of the scale of surveillance.

Future research can be focused on checking the progression of symptom-related search terms over time in order to characterize the clinical course of COVID-19 by means of examining a range of possible search term–based definitions for initial symptom onset. This should be based on various combinations of the earliest peaking search terms and a detailed understanding of the stage of illness and the manifestations of COVID-19 in the local environment and over time. Studies have indicated that the spread and severity of COVID-19 can be affected by local conditions, and search volume data can be a valuable complementary tool for studying potential local variations in disease presentation. Given the numerous limitations of laboratory testing, search data are a valuable complementary tool for the population-scale tracking of pandemics in real time.

#### Principal Results

This study showed that the data obtained from Google Trends searches for Spanish keywords related to COVID-19 (ie, “coronavirus,” “neumonía,” “fiebre,” “tos,” “pérdida de olfato,” “dolor de garganta,” and “diarrea”) correlated with data published by the CNE on the daily incidence of laboratory PCR-confirmed COVID-19 cases, hospitalization, intensive care unit admissions, and deaths from COVID-19, going from R=0.635 for “fatigue” to a maximum of R=0.908 for “fever”. We also found that the Google Trends data correlated with the daily incidence of COVID-19 with an 11-day time lag.

It should be noted that for “fatigue,” the day with the highest correlation was day 36 (ie, the sixth week after the search). Statistically, this is moderately relevant, but considering the high variability in the number of days in incubation, pathogenesis, and generation of an immune response for COVID-19, this relevance may not be so evident when evaluating future COVID-19–positive cases. Consequently, it is possible that fatigue should not be considered as a symptom for assessing and predicting a positive case using Google Trends.

Although we used correlations to examine the possible linear association between search queries and daily COVID-19 incidence, it should be noted that the use of a search engine is voluntary, and self-initiated search queries represent the users who are truly curious or worried about a situation. Thus, we believe that the unobtrusive search behavior of netizens may have resulted in an increase in search volume. The analysis and methods used in this study could aid public health and communication agencies. It is crucial to study this association for the rest of Europe, since other countries, such as Italy, Great Britain, and France, have been affected by the COVID-19 pandemic, and new waves of COVID-19 are foreseeable as long as social distancing measures are relaxed and the winter cold reenters.

This study presents the need for a detailed survey that provides data on the clinical features of COVID-19, prevention strategies, and technological solutions, including search engine data that have been at the forefront of health research. Findings from this study validate and extend previously published works that used Google keywords [5,6,8], and we demonstrate the potential of using Google for monitoring and predicting the evolution of the COVID-19 pandemic. By using Google Trends, this study identified that there is growing interest in COVID-19 worldwide and in countries with a high incidence of SARS-CoV-2 virus infection.

#### Limitations

Our study used Google Trends, which only provides the search behavior of people using the Google search engine. Future studies should consider studying the same topic, but with other search engine platforms to capture a more diverse population of users. The use of an automated program [31] can improve the accuracy of the data collected and analyzed in countries with a high incidence of SARS-CoV-2 virus infection. Furthermore, the selection of keywords plays a very important role in ensuring the validity of our results. Taking into account that this field of research is relatively new, there is no standard method for reporting, resulting in the same meaning of different terms, different meanings of the same term, and different abbreviations. In addition, search data may be subject to socioeconomic, geographic, or other biases inherent in the local digital divide. Lastly, Google Trends does not provide information about the methods used to generate search data and its algorithms. Therefore, other search engines should be investigated. The transfer of conclusions to countries with a low level of internet access should be done with caution.

#### Comparison With Prior Work

By using Google Trends, this study identified that there is growing interest in COVID-19 worldwide, as well as in countries with a high incidence of SARS-CoV-2 virus infection. This study is consistent with previous studies, such as those listed in Table 1, since all of those studies found a positive correlation between searches related to COVID-19 and the evolution of the pandemic. Furthermore, the correlation lag model of these studies’ data series is within the range of our findings.

#### Conclusions

Further research is necessary to determine if the lag detected in our study is related to the results of clinical studies that postulate 97.5% of symptomatic COVID-19 cases develop within 11.5 days after exposure [32]. This 11.5-day adjustment is an improvement over the initial case adjustment date of 15 days. In fact, during the second wave of the pandemic, a 10-day quarantine has been considered sufficient in many places. For visualization purposes, a 10-day moving average would provide slightly clearer plots.

Another priority in the early stages of an emergent pandemic, such as the COVID-19 pandemic, is to characterize the clinical course of symptoms in affected individuals. If population-scale clinical patterns could be ascertained early, it would be beneficial to pandemic tracking, case diagnosis, and treatment. Therefore, we investigated whether internet search data could be used to characterize the clinical course of COVID-19 symptoms over time and provided a search data–based view of the clinical course of the illness.

With regard to future studies, it could be useful to use Pytrends [31], a simple interface for automatically downloading reports from Google Trends. Furthermore, methodologies beyond those first developed [11] must be advanced to approach studies on internet search patterns in a systematic way. For countries where the inflection curve has not yet occurred, a systematic approach can be the most useful if governments monitor the evolution of Google queries in their country to foresee the best use of their hospital systems.

## Abbreviations

CNE: Centro Nacional de Epidemiología
PCR: polymerase chain reaction
RSV: relative search volume
